# Development of diagnostic microsatellite markers from whole-genome sequences of *Ammodramus* sparrows for assessing admixture in a hybrid zone

**DOI:** 10.1002/ece3.1514

**Published:** 2015-05-13

**Authors:** Adrienne I Kovach, Jennifer Walsh, Jordan Ramsdell, W Kelley Thomas

**Affiliations:** 1 Department of Natural Resources and the Environment, University of New HampshireDurham, New Hampshire, 03824; 2 Department of Molecular, Cellular and Biomedical Sciences, University of New HampshireDurham, New Hampshire, 03824

**Keywords:** Admixture, *Ammodramus*, diagnostic markers, hybridization, next-generation sequencing

## Abstract

Studies of hybridization and introgression and, in particular, the identification of admixed individuals in natural populations benefit from the use of diagnostic genetic markers that reliably differentiate pure species from each other and their hybrid forms. Such diagnostic markers are often infrequent in the genomes of closely related species, and genomewide data facilitate their discovery. We used whole-genome data from Illumina HiSeqS2000 sequencing of two recently diverged (600,000 years) and hybridizing, avian, sister species, the Saltmarsh (*Ammodramus caudacutus*) and Nelson's (*A. nelsoni*) Sparrow, to develop a suite of diagnostic markers for high-resolution identification of pure and admixed individuals. We compared the microsatellite repeat regions identified in the genomes of the two species and selected a subset of 37 loci that differed between the species in repeat number. We screened these loci on 12 pure individuals of each species and report on the 34 that successfully amplified. From these, we developed a panel of the 12 most diagnostic loci, which we evaluated on 96 individuals, including individuals from both allopatric populations and sympatric individuals from the hybrid zone. Using simulations, we evaluated the power of the marker panel for accurate assignments of individuals to their appropriate pure species and hybrid genotypic classes (F1, F2, and backcrosses). The markers proved highly informative for species discrimination and had high accuracy for classifying admixed individuals into their genotypic classes. These markers will aid future investigations of introgressive hybridization in this system and aid conservation efforts aimed at monitoring and preserving pure species. Our approach is transferable to other study systems consisting of closely related and incipient species.

## Introduction

Interspecific hybridization is common in nature (Mallet 2005; Abbott et al. 2013), especially among species in early stages of speciation or in secondary contact (Via 2009; Ellegren et al. 2012). Wild hybrids are a mosaic of phenotypes and genotypes, creating challenges for their taxonomic identification and confusion about their conservation status (Stronen and Paquet 2013). Accurate identification of admixed individuals in wild populations aids evolutionary investigations of introgressive hybridization as well as conservation management.

Studies of genetic admixture are most powerful when they use diagnostic species-specific markers, that is markers that are highly differentiated between the two parental species (Moccia et al. 2007; Hohenlohe et al. 2011). Yet, diagnostic markers are infrequent in the genomes of closely related species, and they are rarely found by anonymous marker development approaches. Current sequencing technologies present solutions to the challenges of identifying diagnostic markers, through efficient development of large genomewide panels of SNPs or microsatellite loci. Despite the advent and potential power of large SNP panels, there are many research questions, including those involving genetic hybrid indices, that can be effectively addressed with a carefully selected suite of highly informative microsatellite markers (Guichoux et al. 2011; Wei et al. 2014; Vukosavljev et al. 2015). High-throughput sequencing greatly enhances de novo microsatellite development and results in the low cost recovery of tens of thousands of repeat-containing sequences (Malausa et al. 2011; Reid et al. 2012). Diagnostic marker development can capitalize on this wealth of repeat sequence data to identify markers in the few genomic regions that are differentiated between closely related species. By generating sequencing data from both species' genomes, screening markers for repeat differences can be performed in silico, thereby saving tremendously on the laborious process of screening loci in the laboratory. In this study, we developed such a strategy for comparing the repeat sequences generated by whole-genome shotgun sequencing of two hybridizing avian sister species, to identify a suite of diagnostic markers for high-resolution identification of pure and admixed individuals in an avian hybrid zone.

**Figure 1:**
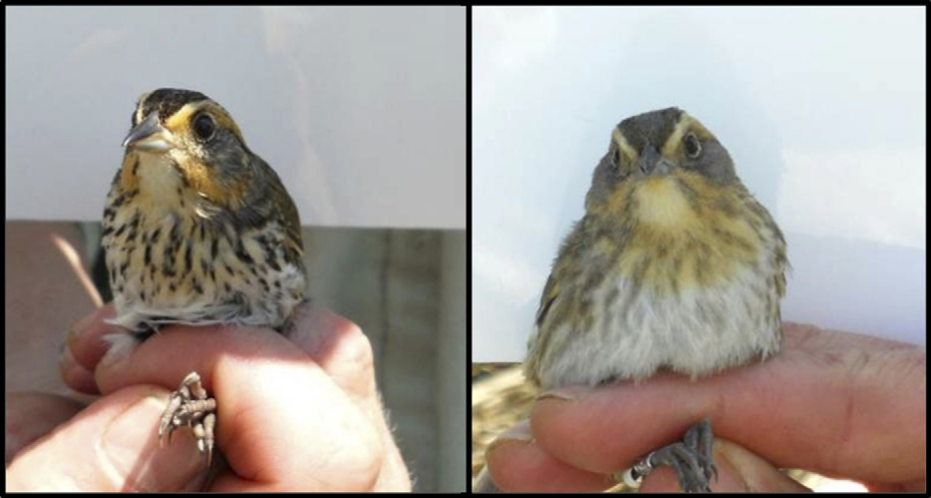
Pure Saltmarsh Sparrow (*Ammodramus caudacutus*) on the left and pure Nelson's Sparrow (*Ammodramus nelsoni*) on the right. The two tidal marsh birds hybridize in an overlap zone along the northeastern Atlantic coast, from northern Massachusetts to southern Maine, USA.

Saltmarsh and Nelson's sparrows (*Ammodramus caudacutus* and *A*. *nelsoni*; Fig.1) belong to a unique group of terrestrial vertebrates that rely primarily or exclusively on tidal marsh habitats (Greenberg et al. 2006a). As such, they are excellent models for studying local environmental adaptation and ecological speciation (Greenberg and Maldonado 2006; Greenberg 2006). They are also species of high conservation priority along the northeastern Atlantic coast of North America, where they breed (U.S. Department of Interior (USDI) 2008). *A. caudacutus* breeds exclusively in coastal marshes from mid-Maine to Virginia, USA (Greenlaw and Woolfenden 2007); it is globally threatened because of its limited range and obligate habitat requirements (IUCN Red List criteria; Birdlife International 2004). *A. nelsoni* has a wider ecological niche, and one of three subspecies (*A. n. subvirgatus*) breeds in tidal marshes, brackish waters, and hay fields from coastal Quebec to northeastern Massachusetts (Greenlaw and Woolfenden 2007; Nocera et al. 2007). These young species diverged ∽600,000 years ago (Rising and Avise 1993), as evidenced by weak genetic divergence (1.2% differentiation at the COI gene and *F*_ST_ of ∽0.15 for neutral microsatellite markers; Shriver et al. 2005; Walsh et al. 2011). They co-occur and hybridize in tidal marshes of the New England coast, where they are now in secondary contact.

Hybrid *A. caudacutus-nelsoni* sparrows are prevalent within the overlap zone and reveal a complex and poorly understood pattern of morphological and genetic introgression (Hodgman et al. 2002; Shriver et al. 2005; Walsh et al. 2011). Currently available microsatellite markers yield low levels of differentiation within and between species (Shriver et al. 2005; Walsh et al. 2012) and lack the resolution to differentiate genotypic classes of admixed individuals (e.g., F1, F2, and backcrossed to each parental species). Difficulties in distinguishing pure and admixed individuals hinder efforts to evaluate the productivity and viability of populations in the hybrid zone, as well as to fully evaluate the geographic extent of introgression. Diagnostic markers are germane for addressing these concerns as well as for investigating patterns and mechanisms of introgressive hybridization.

The aim of this study was to use whole-genome sequence data of *A. caudacutus* and *nelsoni* for de novo development of a suite of species-specific diagnostic microsatellite markers with high resolution for identifying pure and hybrid genotypic classes (F1, F2, and backcrosses). To do so, we identified putative diagnostic markers by in silico comparison of repeat sequences in the two genomes, and we screened 37 of them in the laboratory on individuals of both species. We then developed a panel of the 12 most diagnostic markers, which we found through additional screening to be highly suitable for a genetic hybrid index. We evaluated the power of the markers for accurate assignments of simulated individuals to their appropriate hybrid genotypic classes. Our approach and PERL script for identifying diagnostic repeats between two genomes are readily transferrable to other study systems consisting of closely related and incipient species.

## Materials and Methods

### Sampling and DNA extraction

To obtain samples for marker development, we sampled a total of 120 *A. caudacutus* and *nelsoni* individuals from multiple putatively allopatric (*n* = 48 individuals of each species) and sympatric (*n* = 24 individuals) locations along the northeastern coast of the United States, within and north and south of the species' overlap zone (Table1). Adult sparrows were captured using 12-m mist nests with size 36-mm mesh. Blood samples (10–20 *μ*L) were collected from the brachial vein onto Nobuto blood filter strips (Advantec MFS Inc., Dublin CA). For de novo marker development, two additional females, one *nelsoni* captured from Penobscot, Maine, and one *caudacutus* captured from Middletown, Rhode Island, were blood-sampled for whole-genome sequencing. These individuals were assumed to be “pure” for each parental species, as they were sampled from locations outside of the currently recognized hybrid zone (Hodgman et al. 2002). DNA was extracted from blood samples using a DNeasy Blood Kit (Qiagen, Valencia, CA).

**Table 1:** Sampling locations and sample sizes of *Ammodramus caudacutus* and *A. nelsoni* used in this study. Locations outside of the currently documented overlap zone (considered allopatric populations in this study) are in bold

Sampling Location	Latitude	Longitude	*n*	Sample use
**Lubec, Maine**	44.822	−66.991	9 *nelsoni*	Initial screening/marker characterization
**Columbia Falls, Maine**	44.644	−67.719	9 *nelsoni*	Initial screening/marker characterization
**Narraguagus River – Milbridge, Maine**	44.551	−68.891	9 *nelsoni*	Initial screening/marker characterization
**Penobscot River – Penobscot, Maine**	44.591	−68.859	1 *nelsoni*	Whole-genome sequencing
**Frankfort, Maine**	44.587	−68.858	12 *nelsoni*	Initial screening/marker characterization
**Winterport, Maine**	44.623	−68.854	9 *nelsoni*	Initial screening/marker characterization
Weskeag Marsh – South Thomaston, Maine	44.077	−69.142	1 *nelsoni,* 1 *caudacutus*	Marker characterization
Sheepscot River – Newcastle, Maine	44.065	−69.597	2 *nelsoni*	Marker characterization
Popham Beach – Phippsburg, Maine	43.739	−69.806	1 *nelsoni,* 1 *caudacutus*	Marker characterization
Maquoit Bay – Brunswick, Maine	43.867	−69.988	1 *nelsoni*	Marker characterization
Cousins River – Yarmouth, Maine	43.811	−70.156	1 *nelsoni*	Marker characterization
Saco River – Saco, Maine – Rachel Carson NWR	43.492	−70.391	2 *nelsoni*, 1 *caudacutus*	Marker characterization
Marshall Point – Arundel, Maine – Rachel Carson NWR	43.381	−70.433	1 *nelsoni*	Marker characterization
Eldridge Marsh – Wells, Maine – Rachel Carson NWR	43.292	−70.572	1 *nelsoni,* 1 *caudacutus*	Marker characterization
Kittery Point, Maine – Rachel Carson NWR	43.087	−70.664	1 *caudacutus*	Marker characterization
Lubberland Creek – Newmarket, New Hampshire Great Bay NERR	43.073	−70.903	1 *nelsoni,* 1 *caudacutus*	Marker characterization
Chapman's Landing – Stratham, New Hampshire Great Bay NERR	43.041	−70.924	1 *caudacutus*	Marker characterization
Awcomin Marsh – Rye, New Hampshire	43.006	−70.752	1 *nelsoni,* 1 *caudacutus*	Marker characterization
Hampton Beach, New Hampshire	42.926	−70.806	1 *caudacutus*	Marker characterization
Salisbury, Massachusetts	42.844	−70.822	1 *caudacutus*	Marker characterization
Plum Island – Newbury, Massachusetts (Parker River NWR)	42.774	−70.809	4 *caudacutus*	Initial screening/marker characterization
**Revere, Massachusetts**	42.436	−71.011	5 *caudacutus*	Marker characterization
**Duxbury, Massachusetts**	42.053	−70.681	1 *caudacutus*	Marker characterization
**Waquoit Bay – Mashpee, Massachusetts (Waquoit Bay NERR)**	41.555	−70.506	2 *caudacutus*	Marker characterization
**Prudence Island, Rhode Island**	41.625	−71.321	9 *caudacutus*	Initial screening/marker characterization
**Middletown, Rhode Island – Sachuest Point NWR**	41.488	−71.249	1 *caudacutus*	Whole-genome sequencing
**Narragansett, Rhode Island – John H. Chafee NWR**	41.442	−71.467	9 *caudacutus*	Initial screening/marker characterization
**Shirley, New York – Wertheim NWR**	40.771	−72.889	3 *caudacutus*	Initial screening
**Oceanside, New York – Oceanside Marine Nature Center**	40.622	−73.624	2 *caudacutus*	Initial screening
**North Sea, New York – Scallop Pond Preserve**	40.944	−72.429	2 *caudacutus*	Marker characterization
**Sag Harbor Bay – Noyack, New York**	41.022	−72.306	3 *caudacutus*	Marker characterization
**North Cinder Island – Lido Beach, New York**	40.602	−73.611	3 *caudacutus*	Marker characterization
**Plum Bank Creek – Old Saybrook, Connecticut**	41.269	−72.391	2 *caudacutus*	Marker characterization
**Farm River State Park – East Haven, Connecticut**	41.255	−72.857	2 *caudacutus*	Marker characterization
**Milford, Connecticut**	41.218	−73.035	1 *caudacutus*	Marker characterization
**Watts Island – Niantic, Connecticut**	41.299	−72.219	1 *caudacutus*	Marker characterization
**West River – West Haven, Connecticut**	41.291	−72.945	1 *caudacutus*	Marker characterization

### Genome sequencing and assembly

Illumina TruSeq DNA libraries were generated including electrophoretic, gel-based, manual size selection targeting an average insert size of 300 bp. Whole-genome 100–base pair, paired-end sequencing was performed in two separate lanes on an Illumina HighSeqS2000. This resulted in 213,519,998 and 384,563,744 100-base-pair reads for *A. caudacutus and nelsoni* genomes, respectively.

De Novo assembly of each genome was constructed from the paired reads (after filtering out reads with any ambiguous nucleotides – Ns) using the CLC Genomics Workbench 4.5.1 (CLC Bio, Aarhus, Denmark). Assembly parameters were as follows: kmer size = 26, bubble size = 50, mismatch cost = 2, insertion and deletion costs = 3, length and similarity fractions of 0.5 and 0.8, respectively, and scaffolding set to true. The draft assembly for *A. caudacutus* is comprised of 237,108 contigs (largest contig is 188,803 bp) and the *A. nelsoni* assembly is comprised of 142,556 contigs (largest contig is 442,557 bp). N50 contig sizes are 12,145 and 30,931 bases, with 21X and 37X average coverage for the *A*. *caudacutus* and *A*. *nelsoni* genomes, respectively. Total assembled genome sizes were approximately 1 GB for each species.

### Diagnostic loci identification and primer development

We used the program MSATCOMMANDER version 1.0.8 (Faircloth 2008) to identify repeat motifs (tri- and tetranucleotides) within assembled contigs of the *A. nelsoni* genome that were larger than the N50 contig length. To identify diagnostic repeat sequences, a custom PERL script (Appendix A1) was developed to identify repeat sequences that were common to both species and to compare the repeat numbers between the two genomes. Our script searched the assembled *A. caudacutus* genome for the same 20-base-pair flanking sequences on either side of the repeat regions identified in the *A*. *nelsoni* genome. Reverse complement sequences were similarly searched. Using this filtering process, we identified 1030 tri- and tetranucleotide loci that were common to both genomes. To increase the probability of finding diagnostic markers, we focused on sequences with at least four matching repeats and that differed by 3–10 repeats between species. This resulted in 79 loci; we narrowed this list down further to include only those loci (*n* = 42) that differed by 4–10 repeats. Primers were designed with PRIMER 3 version 0.4.0 (Rozen and Skaletsky 2000), using default parameters, for 37 of these putatively diagnostic loci. To assess the distribution of the 37 loci across the genome, we used BLASTn, with an E value of <1e^−75^ and >80% identity score, to identify the chromosome in the Zebra Finch genome where each repeat sequence was located and annotations when available (Table2). We use the Zebra Finch because it is a well-annotated genome and synteny is high in avian genomes (Warren et al. 2010; Ellegren et al. 2012).

**Table 2:** Description of 34 putatively diagnostic markers screened in 12 *Ammodramus caudacutus* and 12 *A. nelsoni* individuals from allopatric populations. For each locus, columns contain the repeat motif, size in base pairs of the amplified fragments, primer sequences and annealing temperature, number of individuals genotyped (*n*), number of unique alleles (NA), observed (H_o_) and expected (H_e_) heterozygosities, number of alleles shared between the two species, the chromosome location and annotation of the amplified sequence in the Zebra Finch genome, and the GenBank accession number. Loci in bold are the 12 diagnostic markers selected after initial screening

Locus	Repeat motif	Size Range (bp)	Primer sequences (5′–3′)	TA (°C)	*n*	NA	H_O_	H_E_	# of Shared Alleles	Chromosome number (Zebra Finch)	Zebra Finch Annotation	GenBank Accession Number
***Ammo*****001**	ACTC	138–158	F: CTTTCATCCATCCCTGTGCT	63	12^1^	6	0.318	0.374	0^2^	13	Long-chain fatty acid CoA ligase 6	KR011201
			R: AGGTCAAGCCTTGCATCTGT									
*Ammo*002^2^^,^^3^	AAT	194–242	F: GGTGTTAGCAGCCCAGGTAT	60	23	9	0.436	0.684	2	4	NA	KR011202
			R: CCTCAGGAGGTCAGTTTTGC									
***Ammo*****003**	GAT	139–157	F: TGTTTGAGAAACAAAAGCCAAT	60	23	4	0.261	0.403	1	2	mdm2-binding protein	KR011203
			R: CCCATTTCTCTCAAGGACCA									
*Ammo*005	AAAT	184–224	F: TGCCTTTTCCTGTGGAGACT	60	23	9	0.523	0.601	1	2	NA	KR011205
			R: CCTGTCGCTTGCTAATGGAT									
***Ammo*****006**	ACAT	228–264	F: TTCCAGCCCTTTTTGTTGAG	60	23	9	0.648	0.686	0	1	Mitogen-activated protein kinase	KR011206
			R: GCAAGGAAATCAGGCTGTGT									
***Ammo*****008**	GAT	238–250	F: AAGGCAGATGTTCCCAACAC	60	24	3	0.208	0.309	2	1	NA	KR011208
			R: CGCAAACTCCCAGAACTGTA									
*Ammo*009^2^	AAAC	242–270	F: TGGGTGACTTAAGGGTGTCAG	56	22	7	0.545	0.636	2	3	Estrogen-related receptor gamma	KR011209
			R: GGGCTTGAAAAGCTTGTAATTG									
*Ammo*010^4^	AAAC	230–254	F: AGCCCTCATGCAGGTAAGAA	60	22	7	0.275	0.597	3	1	NA	KR011210
			R: TCCAGAGGGTTTGCTCAACT									
*Ammo*011	AAT	206–236	F: GGCACCTCTGACGATCAAAT	60	23	9	0.424	0.503	2	3	NA	KR011211
			R: ATAACAGCAAGACCGCCACT									
***Ammo*****012**^5^^,^^6^	GTT	176–191	F: TTTGAACAATTCCTTCAATGG	56	23	3	0.000	0.243	0	2	NA	KR011212
			R: CAGCATTCCGCAAGTCATAA									
*Ammo*013^2^	ACCT	252–272	F: GAAGCAATGCAGGAGGAAAC	60	23	6	0.254	0.466	1	3	Protein eyes shut homolog	KR011213
			R: CTGAAAATGTGCTTGCCATC									
*Ammo*014	ATT	178–193	F: GAGAAACCTCATTGGGCTTG	60	20	4	0.400	0.350	2	1A	Anoctamin-2	KR011214
			R: GCTTGTGTGCAGGTCTGTGT									
***Ammo*****015**^5^	AGG	242–254	F: TCACCCAAAGGAGGAGTTTG	60	22	4	0.083	0.179	1	3	NA	KR011215
			R: TCCCCTGGGATGTGTAATGT									
***Ammo*****016**[Table-fn tf2-5]^,^^6^	AAT	244–259	F: GCAAAGCATGCACTGACAAT	60	22	4	0.087	0.344	1	2	NA	KR011216
			R: CCTCACCTGCTTTCAACTCC									
***Ammo*****017**	AATC	112–116	F: GCTCTGGAGTGCTGCAAAAT	60	11^1^	2	0.045	0.118	0^1^	1A	Thromboxane-A synthase	KR011217
			R: AGGGTCAAAACAGAGCATGG									
*Ammo*018	ATTT	200–236	F: GGCTCGAAGACCTGGATGTA	60	24	8	0.542	0.569	2	No match	NA	KR011218
			R: AGCCTCAAATCCAACATTCC									
*Ammo*019^5^	ATT	150–171	F: CCTGCAGGAAATGAGAGAGC	60	22	4	0.182	0.432	1	3	NA	KR011219
			R: TGCGCATGAAGTCATAGTCAT									
*Ammo*020	AAT	265–292	F: TTGGTTTCAAAGGAGATTTTTCA	60	23	9	0.867	0.739	6	4	NA	KR011220
			R: GGTCTAATCAAGGTGGCACAA									
*Ammo*021^2^	CTGT	146–166	F: GGGTGGCACAGTCACATTTT	63	24	6	0.917	0.660	2	5	NA	KR011221
			R: GTGTCAAGGTCCACCTGCTT									
*Ammo*022^4^^,^^6^	CCT	239–263	F: TGAGAGTCCTGCAGCCTTG	60	23	7	0.133	0.536	2	No match	NA	KR011222
			R: CAGCAAACACAAAGGTGGAA									
***Ammo*****023**^5^	ATT	213–261	F: GGAACCAGAGAGTCCCACCT	60	22	11	0.500	0.657	1	2	NA	KR011223
			R: AAAGGCTTCTGCATCAGAAAAT									
*Ammo*024	AAAT	268–284	F: TTTCAAAGGTCTGGTACAGCAA	60	22	4	0.364	0.390	2	1	Gamma-aminobutyric acid receptor subunit beta-3	KR011224
			R: CCTCAAGTCCTTTGCCATGT									
*Ammo*025^5^	ATT	183–225	F: GCTTCCCCTTCTTTCCAAGT	60	22	12	0.592	0.780	4	No match	NA	KR011225
			R: CTCCTGGTACGTGCCATTTT									
***Ammo*****027**^2^^,^^3^	AAAG	190–232	F: AAAAGGAAAGCTTCAGTGACAAA	63	22	9	0.592	0.713	1	3	SET and MYND domain-containing protein 3	KR011227
			R: ATTTAAGGGGCTGCTCTTGG									
*Ammo*028	ATCC	228–260	F: GCAGCTGCTTCCTAACCTTG	60	24	9	0.750	0.793	6	2	NA	KR011228
			R: GGCACTTAACGTGGGTTTGT									
*Ammo*029^2^	AATG	116–148	F: TGAAACAAAGGAATTTGGAAAGA	63	20	6	0.450	0.675	3	1	NA	KR011229
			R: CTGGAAAATGCCCAGACACT									
***Ammo*****030**	ATT	243–270	F: GCCAATGAACGTCCTCAAAT	60	20	7	0.438	0.414	1	3	NA	KR011230
			R: GAACAGTGCAGCCAACTTCA									
*Ammo*031^2^^,^^3^	ACT	244–259	F: AAAAGCTAAAACCTTAGACATCAGC	60	23	4	0.174	0.349	2	Z	NA	KR011231
			R: TTCATTTCCTTAGGGAGGAACA									
*Ammo*032^4^	GAT	137–158	F: AAAACCCTAGGGGAAGGACA	63	23	6	0.871	0.637	2	Z	NA	KR011232
			R: ACACACAAGTGGCAGCTGTT									
*Ammo*033^5^	ATCC	262–278	F: TACCAGGAAATGCCACACAA	60	22	5	0.545	0.630	4	No match	NA	KR011233
			R: TGTTCTGCAAGGTGCTATGG									
*Ammo*034	AAT	129–165	F: AGGGAAGAATCTGTACCTGCT	60	21	9	0.864	0.765	4	No match	NA	KR011234
			R: GCAGATGCAGCATAACAAGC									
*Ammo*035^4^	ATCT	161–229	F: ACACCGCAAGCCAAAGTAGT	60	24	11	0.590	0.743	5	1	NA	KR011235
			R: GACCGGGATTTCCATTCATA									
***Ammo*****036**	CTT	189–210	F: TCAGAGGCGTTGTCCTTTCT	60	21	5	0.527	0.528	2	1A	NA	KR011236
			R: TTGAGGAGAAGGGTTGATGG									
*Ammo*037	ATCT	268–312	F: CATGCTGCTTGGACTTCTCA	63	23	10	0.739	0.646	3	3	CUB and sushi domain-containing protein 1	KR011237
			R: TGAGTGATGCTGACCTGTGC									

1 Did not amplify in *A. caudacutus*.

2 Out of Hardy Weinberg Equilibrium (HWE) in *A. nelsoni* at *P* = 0.05.

3 Out of HWE in *A. nelsoni* with Bonferroni correction.

4 Out of HWE in both species at *P* = 0.05.

5 Out of HWE in *A. caudacutus* at *P* = 0.05.

6 Out of HWE in *A. caudacutus* with Bonferonni correction.

### Genotyping and microsatellite characterization

To test the 37 diagnostic loci for amplification, we chose two individuals of each species. Polymerase chain reactions were prepared in 12.5 *μ*L reactions and contained 2 *μ*L of eluted genomic DNA, 0.4 *μ*mol/L of each primer, 2.5 mmol/L MgCl_2_, 5X PCR buffer (Promega, Madison, WI, USA), 0.2 mmol/L of deoxyribonucleotides, and 1 unit of Taq DNA polymerase (Promega). Cycling conditions were as follows: initial denaturation at 94°C for 4 min, followed by 30 cycles of 94°C for 30 sec, 56°–63°C for 45 sec, 72°C for 1 min, and a final extension step at 72°C for 5 min. PCR products were resolved on a 1% agarose gel. Of the 37 primers, 34 consistently amplified the target regions in both species and were used for an initial screening of 24 individuals from eight allopatric marshes (Table1). PCR was repeated with the addition of 0.04 mmol/L of fluorescently labeled ChromaTide Alexa Fluor 488-5-dUTPs (Invitrogen, Life Technologies, Grand Island, NY, USA) to allow for the visualization of amplified products on an automated DNA sequencer (ABI 3130 genetic analyzer, Applied Biosystems, Foster City, CA).

To characterize the diagnostic potential of these 34 loci, we counted the number of alleles shared between the species across the 24 allopatric individuals (Table2). We chose 12 loci with the fewest number of shared alleles and the most variation in the distribution of alleles to screen further as a panel of putatively diagnostic loci. These 12 chosen loci were screened in an additional 96 individuals (36 allopatric and 12 sympatric individuals of each species), using dye-labeled primers (HEX, FAM, or NED) in two multiplex PCRs. The 15 *μ*L polymerase chain reactions contained 3 *μ*L of eluted genomic DNA, 0.1–0.3 *μ*mol/L of each dye-labeled primer, 2.0 mmol/L MgCl_2_, 5X PCR buffer (Promega), 0.1 mmol/L of deoxyribonucleotides, and 1 unit of Taq DNA polymerase (Promega). We used the same cycling conditions described above with a 60°C annealing temperature for all loci. Amplified products were again electrophoresed on an ABI 3130 automated DNA sequencer, and individual genotypes were scored manually using PEAKSCANNER software (ABI).

For the 12 diagnostic loci, the number of private alleles, allele frequencies, and estimates of expected and observed heterozygosities were calculated for allopatric individuals in GENALEX, version 6.41 (Peakall and Smouse 2006). The proportion of shared alleles was calculated for each locus as the number of alleles shared between allopatric *A*. *nelsoni* and *A*. *caudacutus* divided by the total number of alleles. The frequency of the most common allele in each species was calculated in GENALEX. We performed selection tests for the 12 loci using an *F*_ST_ outlier approach (Beaumont and Nichols 1996) in LOSITAN (Antao et al. 2008). Tests of Hardy–Weinberg equilibrium and linkage equilibrium were conducted in GENEPOP, version 4.2 (Raymond and Rousset 1995). Significance was assessed with a Bonferroni correction for multiple tests. Locus-specific *F*_ST_ values were also calculated for all pairwise combinations of allopatric and sympatric sparrows in GENEPOP. We used a Bayesian clustering method implemented in the program STRUCTURE v. 2.3.4 (Pritchard et al. 2000) to assess how membership proportions differed between allopatric and sympatric populations of both species. We ran ten replications with *K* = 2, using the admixture model with correlated allele frequencies and a 100,000 burn-in followed by 100,000 iterations.

### Power assessment of diagnostic marker panel

We assessed the power of the panel of 12 diagnostic markers by evaluating the accuracy of each locus in assigning known individuals to hybrid classes. We simulated 100 genotypes for each of six genotypic classes (pure *A. nelsoni*, pure *A. caudacutus*, backcrossed *A*. *nelsoni*, backcrossed *A*. *caudacutus*, F1 hybrids, and F2 hybrids) using the program HYBRIDLAB 1.0 (Nielsen et al. 2006). Simulated individuals were analyzed using the program NEWHYBRIDS 1.1 BETA (Anderson and Thompson 2002); we used the z and s option to identify the 36 pure individuals of each species as known reference individuals. We ran NEWHYBRIDS using the default options with 200,000 sweeps and a 200,000 burn-in. We calculated mean posterior probabilities of the individuals assigned to each category and the percentage of correctly assigned individuals. Individuals were considered correctly assigned when their true category was the category with the highest posterior probability.

## Results

### Marker development and characterization

Sizes of the repeat regions for the 34 markers ranged from 112 to 284 bp, and loci were variably polymorphic with 2–12 alleles (Table2; see Appendix A2 for allele frequency data). Mean observed and expected heterozygosities ranged from 0.133 to 0.917. Eighteen loci showed significant deviations from Hardy–Weinberg in one or both species at *P* < 0.05, and 6 loci showed deviations in one or both species after Bonferroni correction (*P* < 0.0007; Table2). These deviations are not unexpected and most likely result from a Wahlund effect (Wahlund 1928), given that samples for each species were collected from a diversity of geographic locations, potentially comprised of distinct populations. The number of shared alleles between species ranged from 0 to 6 across the 34 loci. Across the panel of 12 diagnostic loci, no pairs showed significant deviations from linkage equilibrium. Two loci (*Ammo012* and *Ammo015*) were candidates for positive selection.

### Resolution and power of the diagnostic marker panel

**Figure 2:**
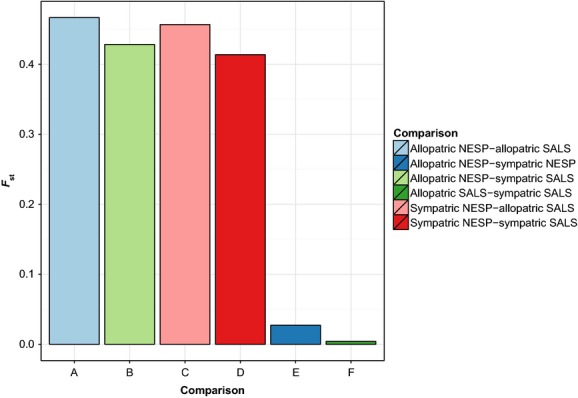
Distribution of *F*_ST_ values across the 12 diagnostic markers for pairwise comparisons of allopatric and sympatric *Ammodramus caudacutus* and *A. nelsoni*.

**Figure 3:**
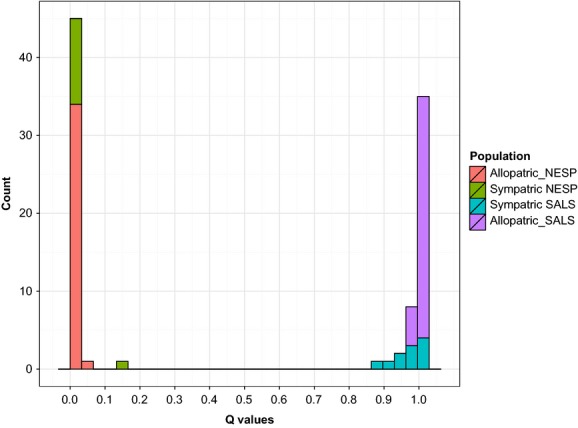
Distribution of Q values from program STRUCTURE for allopatric and sympatric *Ammodramuss caudacutus* and *A*. *nelsoni*. Q values indicate the proportion of the genome attributed to the parental species, with 1 being pure *caudacutus* and 0 pure *nelsoni*.

The proportion of shared alleles between allopatric *A. caudacutus* and *A. nelsoni* at the 12 diagnostic loci ranged from 0.11 to 0.95, with the frequency of most common alleles as high as 1.0 in *A. caudacutus* and 0.984 in *A. nelsoni* (Table3). The number of private alleles ranged from 1 to 12 among allopatric populations. Locus-specific *F*_ST_ values between allopatric *A. nelsoni* and *A*. *caudacutus* ranged from 0.21 to 0.81 with a global *F*_ST_ of 0.46 (Table4). Differentiation between sympatric *A. nelsoni* and allopatric *A. caudacutus* was similar to that of the two allopatric populations; however, differentiation between allopatric *A. nelsoni* and sympatric *A. caudacutus* and between sympatric populations of each species was slightly lower (Fig.2). *F*_ST_ values for within-species comparisons were much lower (0.004 to 0.027 overall; Table4; Fig.2). STRUCTURE Q values (proportion of the genome attributed to the parental species, with 1 being pure *caudacutus* and 0 pure *nelsoni*) for allopatric individuals were above or below the pure species cutoffs of 0.9 and 0.1, respectively. Introgression was apparent in sympatric individuals, however, with slightly lower Q values, including some above/below the pure species cutoffs, especially for sympatric *A*. *caudacutus* (Fig.3).

**Table 3:** Characterization of 12 diagnostic microsatellite markers screened in allopatric *Ammodramus caudacutus* and *A. nelsoni*

Locus	Dye label	Multiplex	Size range (bp)	*n*	NA	H_O_	H_E_	Private alleles	Proportion of shared alleles	Most common allele/frequency in *nelsoni*	Corresponding frequency in *caudacutus*	Most common allele/frequency in *caudacutus*	Corresponding frequency in *nelsoni*
*Ammo*001	Fam	A	118–154	72	9	0.639	0.659	6	0.57	138/0.361	0.00	118/0.556	0.04
*Ammo*003	Fam	B	139–157	71	5	0.454	0.484	2	0.93	154/0.806	0.19	151/0.471	0.06
*Ammo*006	Fam	A	232–264	72	9	0.611	0.696	6	0.49	244/0.403	0.00	260/0.347	0.00
*Ammo*008	Hex	A	238–250	72	4	0.292	0.331	1	0.98	244/0.569	0.01	250/0.944	0.10
*Ammo*012	Hex	B	177–192	64	3	0.183	0.174	2	0.11	177/0.984	0.00	189/0.803	0.00
*Ammo*015	Ned	A	241–256	72	6	0.153	0.185	5	0.42	241/0.972	0.00	253/0.819	0.03
*Ammo*016	Ned	B	245–263	71	6	0.242	0.416	2	0.93	245/0.944	0.03	257/0.386	0.03
*Ammo*017	Hex	A	112–136	72	7	0.500	0.514	2	0.95	116/0.736	0.06	124/0.583	0.15
*Ammo*023	Fam	B	211–256	71	16	0.675	0.711	12	0.65	223/0.403	0.01	214/0.571	0.01
*Ammo*027	Ned	A	188–228	72	10	0.681	0.729	6	0.64	188/0.500	0.10	212/0.306	0.01
*Ammo*030	Hex	B	243–279	71	7	0.333	0.384	6	0.59	264/0.319	0.00	243/1.00	0.18
*Ammo*036	Ned	B	191–215	71	9	0.566	0.550	7	0.34	191/0.764	0.00	194/0.443	0.03

**Table 4:** Locus-specific and overall *F*_ST_ values for all pairwise comparisons of allopatric and sympatric *Ammodramus caudacutus* and *A. nelsoni* using the panel of 12 diagnostic microsatellite markers developed in this study

Locus	*F*_ST_					
	Allopatric *nelsoni*/Allopatric *caudacutus*	Allopatric *nelsoni*/Sympatric *caudacutus*	Allopatric *caudacutus*/Sympatric *nelsoni*	Sympatric *nelsoni*/Sympatric *caudacutus*	Allopatric *nelsoni*/Sympatric *nelsoni*	Allopatric *caudacutus*/Sympatric *caudacutus*
*Ammo*001	0.3033	0.2592	0.4037	0.3707	0.0703	0.0027
*Ammo*003	0.4019	0.3946	0.3653	0.3477	−0.0312	−0.024
*Ammo*006	0.281	0.2237	0.2524	0.1813	−0.0234	−0.0182
*Ammo*008	0.6245	0.5498	0.6262	0.5204	0.0078	−0.0326
*Ammo*012	0.819	0.7123	0.6915	0.4454	0.0632	0.1041
*Ammo*015	0.8073	0.849	0.7748	0.8202	−0.0387	0.0111
*Ammo*016	0.5586	0.6217	0.4856	0.5076	−0.0183	0.0202
*Ammo*017	0.3968	0.4448	0.2555	0.2814	0.0056	−0.0213
*Ammo*023	0.2629	0.2185	0.1842	0.1371	0.0169	−0.0135
*Ammo*027	0.2144	0.2421	0.3734	0.489	0.1711	−0.0027
*Ammo*030	0.5198	0.3685	0.694	0.4561	0.0006	0.064
*Ammo*036	0.4352	0.465	0.4172	0.4614	−0.0241	0.0166
Overall	0.4667	0.4282	0.4567	0.4137	0.0272	0.004

Based on the 12 diagnostic microsatellite markers, NEWHYBRIDS assigned 92% of all the simulated individuals to their true category. Assignment accuracies varied for the categories, ranging from 76% (F2) to 100% (pure *A*. *caudacutus*), with posterior probabilities for the correctly assigned categories ranged from 0.75 (F2) to 0.991 (*A*. *caudacutus*; Table5). Pure individuals had the highest percentage of correct assignments with 98% (*A. nelsoni*) and 100% (*A. caudacutus*) of individuals correctly assigned with posterior probabilities of 0.947 and 0.991, respectively. F1 individuals were also assigned with high accuracy (97% and posterior probability of 0.936). F2 individuals were the most difficult to assign, especially with respect to distinguishing them from backcrossed individuals, with 76% of individuals correctly assigned with a mean posterior probability of 0.75. For backcrossed individuals, 91% were assigned at nearly identical mean posterior probabilities of 0.844 and 0.843. The majority of misassignments were between backcrossed and F2 individuals. There were no instances where backcrossed *A*. *nelsoni* were assigned as backcrossed *A*. *caudacutus* and vice versa.

**Table 5:** Power assessment of the panel of 12 diagnostic markers for assigning simulated sparrow individuals to pure, F1 hybrid, F2 hybrid, and backcrossed (BC) categories, using NEW HYBRIDS. For each genotypic class, the mean posterior probabilities across 100 simulated individual assignments are reported, and the Accuracy column reports the proportions of individuals correctly assigned to each category (individuals were defined as correctly assigned when their true category was the category with the highest posterior probability in the NEW HYBRID assignment)

True category	Assigned category: mean posterior probabilities						
	Pure *nelsoni*	Pure *caudacutus*	F1 Hybrid	F2 Hybrid	BC *nelsoni*	BC *caudacutus*	% Accuracy
Pure *nelsoni*	0.947	0.000	0.000	0.000	0.052	0.000	98
Pure *caudacutus*	0.000	0.991	0.000	0.000	0.000	0.008	100
F1 Hybrid	0.000	0.000	0.936	0.033	0.008	0.021	97
F2 Hybrid	0.004	0.000	0.040	0.750	0.120	0.071	76
BC *nelsoni*	0.040	0.000	0.014	0.090	0.844	0.000	91
BC *caudacutus*	0.000	0.019	0.053	0.083	0.000	0.843	91

## Discussion

While current sequencing technologies afford the potential for generating tens of thousands of genomewide markers for population genomics research (Davey et al. 2011), not all research and conservation applications will require genomewide data (Allendorf et al. 2010). For such applications, including research questions focused on discerning processes for closely related individuals – such as dispersal, kinship, population structure, and admixture – an informative panel of microsatellite markers will remain a valuable tool (Guichoux et al. 2011; Wei et al. 2014; Vukosavljev et al. 2015). In our case study of an avian hybrid zone, we highlight the utility of a carefully selected, high-resolution panel of microsatellite markers for discriminating genotypic classes of pure and admixed individuals. Our strategy for diagnostic marker discovery via in silico screening for microsatellite repeat differences in two species' genomes eliminates the laborious process of manually screening markers in the laboratory. As such, this efficient and highly effective approach should prove useful for other studies requiring diagnostic microsatellite markers for closely related species.

From whole-genome sequence data, we identified 34 polymorphic and diagnostic or partially diagnostic microsatellite markers that amplified in both *A. caudacutus* and *A. nelsoni*. We developed a panel of the 12 loci with the fewest shared alleles between species. All markers in this diagnostic panel amplified consistently using the same routine PCR conditions, making them highly conducive for multiplexing. We demonstrated the power of these loci for the identification of pure and admixed individuals in this avian hybrid zone.

After screening the 12 diagnostic markers on 96 sparrows from allopatric and sympatric sites, we found them to be highly informative for species discrimination. This panel of loci had high resolution for classifying pure and admixed individuals into their genotypic classes. The markers were most powerful for distinguishing among F1, backcrossed, and parental groups (with 91–100% accuracy), while F2s were difficult to distinguish from either F1 or backcrossed groups (76% accuracy). For all loci, the most common allele differed between the two species; this allele was typically rare (<0.05%) in the other species. Only one locus, *Ammo030*, showed a fixed allele in either species – with a single allele in *A. caudacutus* and three private alleles in *A. nelsoni*. An additional four markers had an allele with >90% frequency in one of the two species. While most of the markers exhibited a relatively large portion of shared alleles between species (0.11–0.98), allele frequency distributions differed strongly between the species, and all loci had at least one private allele. Locus-specific *F*_ST_s indicated strong divergence (ranging from 0.2144 to 0.819, with overall *F*_ST_ = 0.4667) between allopatric populations of each species. In comparison, anonymous neutral loci yielded a between-species *F*_ST_ of 0.15 (Shriver et al. 2005).

The lack of microsatellite loci with fixed differences between the two species is notable, given our whole-genome approach. By comparing all of the microsatellite repeats identified from whole-genome shotgun sequencing, we only found 79 loci to differ in repeat numbers between the two genomes (and of these, only 42 met our criteria of differing by four or more repeats). Our resulting panel of 12 diagnostic loci therefore likely represents the largest microsatellite differences across the genomes of these two species. The overall similarity in microsatellite repeats between the two species exemplifies their close relationship as recently diverged sister species and suggests that high genetic similarity is characteristic not only at the mitochondrial level (Rising and Avise 1993; Walsh et al. 2011), but also potentially on a genomewide level. This finding gives insight into genomic similarity of hybridizing taxa and highlights the challenges of identifying diagnostic markers for recently diverged species, as well as the utility of whole-genome sequencing in high-resolution marker development.

The elevated divergence of the diagnostic panel relative to neutral loci previously used in this system (Shriver et al. 2005; Walsh et al. 2012) suggests that these loci may be under selection (Storz 2005; Strasburg et al. 2012). Selection tests identified two of the markers to be under selection in this dataset; however, further research with more targeted sampling schemes may identify additional selected loci. This is supported by the fact that 10 of the 34 (6 of 12 diagnostic) loci aligned with an annotated protein-coding region of the Zebra Finch genome (Table2). These markers may be associated with a region of the genome with a functional role that diverges between the two species; accordingly, these may be important portions of the genome with respect to speciation. As allelic changes induced by natural selection occur faster than those due to neutral processes (Nei 1987), high-resolution gene-associated markers are more powerful than neutral markers for applications that require assigning individuals to distinct population or species groupings (Nielsen et al. 2009, 2012).

The pattern of between-species divergence that we found using the 12 diagnostic markers in this study was fairly consistent across sympatric and allopatric populations. While *F*_ST_s were highest for allopatric comparisons of the species, they were only slightly lower for comparisons that included sympatric populations, suggesting divergence at these loci is maintained in the face of interspecific gene flow in the hybrid zone (Walsh et al. 2011, 2015). These markers therefore appear to be associated with gene regions that do not introgress freely between the two species. For within-species comparisons, the *F*_ST_s are slightly lower within *A*. *caudacutus* than within *A*. *nelsoni*, supporting the hypothesis that introgression is biased in the direction of the *A*. *caudacutus* genome (Shriver et al. 2005; Walsh et al. 2011).

The low within-species divergence we found in this study is an expected outcome, especially for diagnostic markers. *F*_ST_ among *A. caudacutus* in this study is similar to that previously reported by Walsh et al. (2012) using anonymous neutral loci. Despite high levels of gene flow, Walsh et al. (2012) found evidence for fine-scale population structure within *A*. *caudacutus*. The sampling scheme in the current study, however, was not designed for evaluating within-species population structure, as pooling across many geographically separate sympatric or allopatric marshes likely masks some of the underlying population differentiation. Nonetheless, the higher within-species *F*_ST_ found in *A. nelsoni* compared to *A. caudacutus* suggests that a finer scale population genetic structure may be characteristic of the former. More pronounced population structure in *A. nelsoni* relative to *A. caudacutus* is consistent with differences in the species' distributions and demography – *nelsoni* typically occur in smaller numbers in small marshes that tend to be more spatially disjunct than the larger, more continuous coastal marshes typically occupied by *caudacutus* (J. Walsh and A. Kovach, pers. obser.). These are the first population genetic data collected on *A. nelsoni*; future research with a more robust sampling scheme is warranted to characterize population genetic structure in this species. The preliminary data in this study suggest that these markers will be useful for such within-species population comparisons.

In conclusion, our comparative, whole-genome approach has proven useful for identifying high-resolution diagnostic markers in sister species with high genetic similarity. This approach is superior to anonymous marker development, not only because it enables pinpointing species-specific differences, but also because it links the markers to large contigs that can be mapped to genomic regions. The markers identified in this study will aid future research that requires distinguishing pure and admixed individuals in the *A. caudacutus – nelsoni* hybrid zone, as doing so from morphology alone is unreliable (Walsh et al. 2011, 2015). A hybrid index based on 12 diagnostic microsatellite markers provides an inexpensive and simple genetic assay. This diagnostic assay for hybrid identification will prove valuable in efforts that seek to track shifts in species distributions, which is of particular relevance to the conservation of threatened *A. caudacutus* populations (Shriver et al. 2005; Walsh et al. 2011). The diagnostic marker panel will also be useful for studies of evolutionary ecology, such as providing insight into the rates and direction of introgression and estimates of the width and center of the hybrid zone (Barton and Gale 1993). Our marker development approach is easily transferable to other studies, and we provide our PERL script for comparing repeat sequences of two genomes as an appendix.

## Data Accessibility

Sequences of the 34 microsatellite loci are deposited in GenBank (Accession numbers in Table2). Raw Illumina reads are deposited in the Short Read Archive (SRS893469 and SRS897222). Our PERL script for screening repeat sequences across the two genomes is provided as Appendix A1, and allele frequencies for the panel of 12 diagnostic loci are in Appendix A2. Genotypes of the 96 individuals, simulated genotypes used to evaluate the marker panel, and the assembled N50 contigs of *A. nelsoni* and *A. caudacutus* are deposited in Dryad (http://dx.doi.org/10.5061/dryad.f8m84).

## Appendix 1 Perl script for screening diagnostic microsatellite sequences between two genomes

This script takes the microsatellite loci identified from one dataset with MSATCOMMANDER and screens the repeat sequences against a fasta file of sequences (in this case from a second genome) to pull out those loci common to both datasets that differ in repeat number between the two genomes. The resulting output contains information on the number of repeats for each locus in each dataset. This output can then be filtered in a spreadsheet to thresholds according to the study design (e.g., loci that differ by >4 repeats).

Reference: Kovach, A.I., J. Walsh, J. Ramsdell, and K. Thomas. Development of Diagnostic Microsatellite Markers from Whole Genome Sequences of Ammodramus Sparrows for assessing admixture in a hybrid zone.

#!/usr/bin/perl

#Title: Microsat Scanner

#Version: 1.5

#Author: Jordan Ramsdell

#Use: Allows the user to import microsat pattern data from MSATCOMMANDER

# And compare these patterns to data found in a fasta file.

# Exports results to csv format.

#Documentation: This script uses the output from Msat commander to take the

#flanking nucleotides from either side of the microsatellites. This is then used

#to find a potential match in the target fasta file, under the assumption that

#the flanking sequences are identical.

#The reverse-compliment of the flanks are also searched, in case the

#microsatellite is found on the 3′ to 5′ end.

#USAGE: perl MicroSatScan.pl -msat <input.msat> -source <input.fasta> -target <input.fasta> -flank_length <int, default 20 >  -out <output.csv>

#Options:

#-msat <input.msat> Name of the msat commander file to use with this program.

#-source <input.fasta> Name of the fasta file that msat was run on, used to extract flanking sequences.

#-target <input.fasta> Name of the target fasta file to scan through with flanking sequences.

#-flank_length <integer> Specifies the number of flanking nucleotides used in the regex search.

#-out <output.csv> Name of the comma delimited file to create after finishing scan

#The output is in a comma-separated format with the following columns:

#[Clone]: The contigs from the original fasta file that msat commander was run on.

#[Startbp]: The starting location of the microsatellite within the source contig.

#[Repeat]: The number of times the microsatellite repeats within the source contig.

#[Endbp]: The ending location of the microsatellite within the source contig.

#[Type]: The type of repeat

#[Comments]: Whether or not this repeat was found on the forward or reverse strand in the source.

#[MatchContig]: The target contig where the nucleotide repeat matched (based on flanking regions).

#[MatchRepeats]: How many times the microsatellite repeats within the matching contig.

#[Difference]: The difference in the amount of times the microsatellite repeats in the source contig compared to the target contig.

#[MatchSeq]: Displays the nucleotide sequence found between the flanking regions within the matching target contig.

#[MatchStart]: The starting location of the microsatellite within the matching target contig

#[MatchEnd]: The ending location of the microsatellite within the matching target contig

#[MatchLength]: The total length of the matching sequence within the target contig

#[Type]: Type 0 indicates that there was a change in expected length, likely due to the insertion or deletion of a single bp.

# Type 1 (what we're probably more interested in) means that this sequence differs only in the number of microsatellites it has.

use Getopt::Long;

use Pod::Usage;

use strict();

use warnings();

#Variables

my $fasta;

my $source;

my $target;

my $debug;

my $sett;

my $input;

my $output;

my $msat;

my $append;

my $msat_file;

my $source_file;

my $target_file;

my $flank_length;

#Get arguments

GetOptions( “msat=s” => \$msat_file,

“source=s” => \$source_file,

“target=s” => \$target_file,

“flank_length=i” => \$flank_number,

“out=s” => \$output_name)

or die(pod2usage(1));

#Report errors

die pod2usage(“\nError: Please specify -msat file!\n”) if($msat_file eq “”);

die pod2usage(“\nError: Please specify -source file!\n”) if($source_file eq “”);

die pod2usage(“\nError: Please specify -target file!\n”) if($target_file eq “”);

die pod2usage(“\nError: Please specify -out name!\n”) if($output_name eq “”);

#Default flank length

$flank_number ¦= 20;

#Open Msat file

{

open(MSAT, $msat_file) ¦¦ die “Couldn't open -msat file: $msat_file”;

$msat = <MSAT>;

close MSAT;

}

print “Successfully imported MSAT data.\n\nImporting Source FASTA file.\n”;

#Import Source Fasta File

{

local $/ = undef;

open(SOUR, $source_file) ¦¦ die “Couldn't open -source file: $source_file”;

$source = <SOUR>;

close SOUR;

}

print “Successfully imported Source data.\n\nImporting Target FASTA file.\n”;

#Import Target Fasta File

{

local $/ = undef;

open(TARG, $target_file) ¦¦ die “Couldn't open -target file: $target_file”;

$target = <TARG>;

close TARG;

}

print “Successfully imported Target data.\nFiltering data.\n”;

#Clean up data and prepare header

$target =∽ s/\n//g;

$msat =∽ s/”“[.\n\s]*//g;

my @linesplit = split(“\r”, $msat);

$output = shift (@linesplit);

$output.= “,MatchContig,MatchRepeats,Difference,

MatchSequence,MatchStart,MatchEnd,MatchLength,Type\r”;

print “Data filtering complete. Beginning search.\n\n\n”;

#Begin searching for matches in target sequence

while ($#linesplit != -1)

{

my $element = shift @linesplit;

my @split = split(“,”, $element);

my @reference = @split;

my $contig = shift @split;

#Find Contig index match in source

my $cindex = index ($source, $contig.“\n”);

if (($cindex != -1) && ($split[0] ne “No repeats found” or “”))

{

my $stop = index ($source, “>”, ($cindex + 1));

#Extract the sequence (if it's the last contig, we just extract to the end)

my $sequence;

if ($stop != -1)

{

$sequence = substr ($source, $cindex + length($contig),

($stop - ($cindex + length($contig))));

}

else {$sequence = substr ($source, $cindex + length($contig));}

my $startbp = shift @split;

my $repeat = shift @split;

my $endbp = shift @split;

$sequence =∽ s/\n//g;

my $extract = substr ($sequence, $startbp - 1, ($endbp - $startbp));

my $flank_left = substr ($sequence, $startbp - 1 - $flank_number,

$flank_number);

my $flank_right = substr ($sequence, $endbp - 1, $flank_number);

#Extract repeat nucleotides and number of times they occur.

$repeat =∽ m/\Q(\E(.*)\Q)\E\^(\d{1,3})/;

my $repeat_source = $1;

my $times_source = $2;

#Now do a regex lookup on the target fasta file

my $regex = “(?:$flank_left)([ATCGN]{1,200}?)(?:$flank_right)”;

my $flank_left2 =  reverse($flank_left); $flank_left2 = ∽ tr/ATCG/TAGC/;

my $flank_right2 =  reverse($flank_right); $flank_right2 = ∽ tr/ATCG/TAGC/;

my $regex2 =  “(?:$flank_right2)([ATCGN]{1,200}?)(?:$flank_left2)”;

print “Searching Contig: “.$contig.”\nPattern: “.$repeat.”\n”;

my $reverse = 0;

#Is this match different? If so, send to output. Otherwise, throw out match.

if ((($target =∽ m/(?:$flank_left)/g) ¦¦

(($target =∽ m/(?:flank_right2)/g) && (++$reverse))))

{

my $snippet = substr($target, (pos($target) - 1000), 2000);

if (($snippet =∽ m/(?:$regex)/g) ¦¦

(($snippet =∽ m/(?:$regex2)/g) && (++$reverse)))

{

my $matched_repeat = $1;

my $reverse_repeat = reverse ($matched_repeat);

$reverse_repeat =∽ tr/ATCG/TAGC/;

if (($matched_repeat eq $extract) ¦¦

($reverse_repeat eq $extract)){$output.= join(“,”, @reference).”\r”;}

if (($matched_repeat ne $extract) && ($reverse_repeat ne $extract))

{

print “Located difference in pattern:\n”.”--------------------\n”;

#Something's different in this sequence.

$output.= $contig.“,”.$startbp.“,”.$repeat.“,”.$endbp.“,”.shift(@split).”,”.shift(@split);

my $pos = pos($target);

print “Interior Seqeunce: “.$matched_repeat.”\n”;

#Figure out which contig this is on.

my $contig_start = rindex($target, “>”, $pos);

print “CONTIG START: $pos\n\n\n”;

pos($target) = $contig_start;

$target =∽ m/(?:[ATCGN])/g;

my $contig_end = pos($target);

#Extract name of the contig that had the match in the target fasta file

my $contig_name = substr($target, ($contig_start + 1), $contig_end - $contig_start - 2);

$output.= “,”.$contig_name;

#Now where is this repeat located in the contig?

my $repeat_start = $pos - $contig_end - length($matched_repeat.$flank_right) + 2;

my $repeat_end = $pos - $contig_end - length($flank_right) + 2;

print “Target Contig Name: “.$contig_name.”\n”;

print “\nRepeat Start: “.$repeat_start.”\nRepeat End: “.$repeat_end.”\n”;

#Figure out where the repeat differs.

my $times_target = 0;

if ($reverse == 1){$repeat_source = reverse($repeat_source); $repeat_source =∽ tr/ATCG/TAGC/;}

$times_target++ while $matched_repeat =∽ /(?:$repeat_source)/g;

$output.= “,”.$times_target;

my $repeat_dif = $times_target - $times_source;

$output.= “,”.$repeat_dif.”,”.$matched_repeat.”,”.$repeat_start.”,”.$repeat_end.”,”.length($matched_repeat);

#Did a bp change, or was a whole repeat inserted/deleted?

if (length($matched_repeat) != (length($repeat_source) * $times_target)){$output.=“,0\r”;}

else {$output.=“,1\r”;}

print “Repeated: “.$times_target.” times.\nDifference from Original: “.$repeat_dif.”\n--------------------\n”;

}

}

}

else {$output.= join(“,”, @reference).”,No Match\r”;}

print “\nRemaining Lookups: “.$#linesplit.”\n\n\n”;

}

}

print “\n\nSearch complete.\nExporting data to: “.$output_name;

open FILE, “>“.$output_name or die $!;

print FILE $output;

close FILE;

__END__

=head1 NAME

sample - Using GetOpt::Long and Pod::Usage

=head1 SYNOPSIS

perl MicroSatScan.pl -msat <input.msat> -source <input.fasta> -target <input.fasta> -flank_length <int, default 20 >  -out <output.csv>

Options:

-msat <input.msat> Name of the msat commander file to use with this program.

-source <input.fasta> Name of the fasta file that msat was run on, used to extract flanking sequences.

-target <input.fasta> Name of the target fasta file to scan through with flanking sequences.

-flank_length <integer> Specifies the number of flanking nucleotides used in the regex search. Default is 20.

-out <output.csv> Name of the comma delimited file to create after finishing scan.

=head1 DESCRIPTION

B<This program> will read the given input file(s) and do something

useful with the contents thereof.

=cut

## Appendix A2 Allele frequencies for the 12 diagnostic loci screened in 36 *Ammodramus caudacutus* and *A. nelsoni* sampled from outside of the known overlap zone. Diagnostic alleles (found only in one species) are indicated in bold, and the most common allele in each species is indicated with an asterisk

**Table:**

Locus	Allele/n	Allopatric SALS	Allopatric NESP
*Ammo*001	118	0.556*	0.042
	122	0.389	0.042
	126	0.056	0.028
	134	0.000	**0.056**
	138	0.000	**0.361***
	142	0.000	**0.250**
	146	0.000	**0.097**
	150	0.000	**0.097**
	154	0.000	0.028
*Ammo*006	232	0.000	0.014
	236	0.014	0.403*
	240	0.000	**0.083**
	244	0.000	**0.403***
	248	0.000	**0.056**
	252	0.278	0.028
	256	0.250	0.014
	260	**0.347***	0.000
	264	**0.111**	0.000
*Ammo*017	112	0.014	0.083
	116	0.056	0.736*
	120	0.028	0.014
	124	0.583*	0.153
	128	0.222	0.014
	132	**0.056**	0.000
	136	0.042	0.000
*Ammo*008	238	0.014	0.333
	244	0.014	0.569*
	247	0.028	0.000
	250	0.944*	0.097
*Ammo*027	188	0.097	0.500*
	192	0.000	0.042
	196	0.000	**0.292**
	200	0.000	**0.125**
	208	0.028	0.000
	212	0.306*	0.014
	216	**0.139**	0.000
	220	0.153	0.014
	224	0.181	0.014
	228	**0.097**	0.000
*Ammo*015	241	0.000	**0.972***
	244	0.014	0.000
	247	**0.069**	0.000
	250	**0.083**	0.000
	253	0.819*	0.028
	256	0.014	0.000
*Ammo*003	139	0.329	0.014
	142	0.014	0.000
	151	0.471*	0.056
	154	0.186	0.806*
	157	0.000	**0.125**
*Ammo*023	211	**0.100**	0.000
	214	0.571*	0.014
	217	**0.114**	0.000
	220	0.143	0.014
	223	0.014	0.403*
	226	0.057	0.083
	229	0.000	0.014
	232	0.000	**0.125**
	235	0.000	0.028
	238	0.000	**0.056**
	241	0.000	0.014
	244	0.000	0.042
	247	0.000	0.028
	250	0.000	0.042
	253	0.000	0.042
	256	0.000	0.097
*Ammo*030	243	1.000*	0.181
	249	0.000	**0.125**
	264	0.000	**0.319***
	267	0.000	**0.278**
	270	0.000	0.014
	273	0.000	**0.056**
	279	0.000	0.028
*Ammo*036	191	0.000	**0.764***
	194	0.443*	0.028
	197	0.029	0.000
	200	**0.129**	0.000
	203	**0.086**	0.000
	206	**0.229**	0.000
	209	**0.071**	0.000
	212	0.014	0.194
	215	0.000	0.014
*Ammo*016	245	0.029	0.944*
	251	**0.100**	0.000
	254	0.300	0.014
	257	0.386*	0.028
	260	0.157	0.014
	263	0.029	0.000
*Ammo*012	177	0.000	0.984
	189	0.803	0.000
	192	0.197	0.016
